# Correlation of Radiographic and High-Resolution Computed Tomography (HRCT) Features in Lower Lobe Tuberculosis With Microbiological Findings: A Study in the Konaseema Region

**DOI:** 10.7759/cureus.85976

**Published:** 2025-06-13

**Authors:** Bharath Kakileti, Deborah Purushottam M, TSSL Pratyusha, Padmaja N

**Affiliations:** 1 Department of Radiology, Konaseema Institute of Medical Sciences and Research Foundation, Amalapuram, IND; 2 Department of Microbiology, Konaseema Institute of Medical Sciences and Research Foundation, Amalapuram, IND; 3 Department of Pulmonology, Konaseema Institute of Medical Sciences and Research Foundation, Amalapuram, IND

**Keywords:** high-resolution computed tomography, lower lobe tuberculosis, microbiological correlation, pulmonary tuberculosis, radiographic patterns

## Abstract

Objective: This study aimed to evaluate the radiographic, high-resolution computed tomography (HRCT), and microbiological characteristics of pulmonary tuberculosis (PTB) in general, with a focused correlation analysis in a subset of patients with lower lobe tuberculosis (LLTB), to aid in the accurate diagnosis and clinical differentiation of this atypical presentation.

Methods: A prospective observational study was conducted in the pulmonology, radiology, and microbiology departments of Konaseema Institute of Medical Sciences and Research Foundation, Andhra Pradesh. The study period was from December 2022 to June 2024. The study included 40 confirmed cases of LLTB, which were identified based on microbiological and radiological findings. HRCT and chest radiographs were analyzed for characteristic patterns. At the same time, microbial confirmation was obtained through Ziehl-Neelsen (ZN) staining, GeneXpert MTB/RIF assay (Cepheid, Sunnyvale, CA, USA), and culture on Löwenstein-Jensen (LJ) medium (HiMedia Laboratories Pvt. Ltd., Mumbai, India). Spearman's correlation was used to assess the correlation between radiological findings and microbiological confirmation.

Results: Patchy opacities were the most frequently observed radiological abnormality, identified in 37 (50.0%) out of 74 patients, followed by homogeneous consolidation in 31 (41.9%) and cavitation in 14 (18.9%) cases. Nodular opacities and para-pneumonic effusion were noted in nine (12.2%) and eight (10.8%) patients, respectively, reflecting the heterogeneous imaging patterns associated with LLTB. Sputum smear microscopy for acid-fast bacilli (AFB) yielded positive results in 33 (18.2%) cases, while bronchoalveolar lavage (BAL) confirmed tuberculosis in 12 (6.6%) patients. HRCT findings contributed to the diagnosis in 16 (8.8%) cases. Additionally, nine (5.0%) cases of non-resolving pneumonia and 11 (6.1%) cases presenting with hemoptysis underscored the diagnostic complexity of lower lobe involvement.

Conclusion: In conclusion, LLTB shows atypical radiological features, often mimicking pneumonia, with patchy opacities and consolidation. Cavitation is a key diagnostic indicator due to its strong microbiological correlation. A combined clinical, radiological, and microbial approach is essential for accurate diagnosis.

## Introduction

Tuberculosis (TB) remains a primary global health concern, with approximately nine million new active cases reported each year and an estimated 1.7 million deaths annually [1]. The burden of TB is exceptionally high in low-income regions such as Asia, Africa, and South America, which together account for nearly 95% of all TB cases and 98% of TB-related deaths [2]. In Southeast Asia alone, about 44% of the population is affected [2].

Accurate diagnosis of active TB mainly relies on detecting acid-fast bacilli (AFB) through microbiological tests. The sensitivity of sputum smear for AFB detection ranges from 46% to 74%, while sputum culture sensitivity can vary widely from 2% to 95%, depending on disease stage and bacterial load [3]. National data indicate that only 10% to 22% of adults with inactive pulmonary TB tested positive on a sputum smear [4].

Chest X-ray is commonly used as the first-line imaging tool for pulmonary TB because it is affordable, widely available, and associated with low radiation exposure. However, its diagnostic accuracy is limited, around 34% for primary TB and 59% for post-primary TB [5]. In comparison, high-resolution computed tomography (HRCT) offers greater sensitivity for detecting subtle parenchymal changes, early disease activity, and miliary nodules [6]. Studies have indicated that HRCT has a sensitivity and specificity of 88%, with an overall diagnostic accuracy of 88% in identifying active TB [7].

HRCT is also more effective in detecting cavitary lesions, especially in cases with extensive fibrosis or distorted lung architecture [7]. Research has demonstrated a correlation between the number of AFB seen in sputum and the type of morphological abnormalities observed on HRCT [8]. Although many international studies have reported typical HRCT findings in pulmonary TB, limited regional data are available, particularly concerning local populations [7-9].

Lower lobe tuberculosis (LLTB), though less common, presents a particular diagnostic challenge due to its atypical imaging features, which can mimic other lung conditions such as bacterial pneumonia, malignancy, or interstitial lung disease, resulting in misdiagnosis or delayed treatment initiation [10]. In this context, the present study aimed to evaluate the radiographic, HRCT, and microbiological characteristics of pulmonary tuberculosis (PTB) in general, with a focused correlation analysis in a subset of patients with LLTB, to support accurate diagnosis and better recognition of this uncommon presentation.

## Materials and methods

Study design

This prospective observational study was conducted over 1.5 years, from December 2022 to June 2024, in the Departments of Microbiology and Radiodiagnosis at the Konaseema Institute of Medical Sciences and Research Foundation, Andhra Pradesh. All clinically diagnosed PTB cases during the study period were included. A focused correlation analysis was conducted on a subset of patients with predominant LLTB, identified through imaging and microbiological confirmation. The study aimed to evaluate the overall radiographic and microbiological characteristics of PTB and assess the specific correlation between microbiological results, radiographic patterns, and HRCT features in LLTB. Approval from the Institutional Review Board (IRB) of Konaseema Institute of Medical Sciences and Research Foundation (approval no. 83/02.12.2022) was obtained before the study initiation. Informed consent from patients was unnecessary, as the data were retrospectively gathered from the departmental database, with all patient identifiers removed to maintain confidentiality.

Sample size calculation

The sample size was calculated using the standard formula for prevalence studies: $n = (Z&sup2; &times; p &times; (1 - p)) / d&sup2;$, where n is the required sample size, Z represents the Z-score for a 95% confidence level (1.96), p is the estimated prevalence of LLTB (assumed to be 10% or 0.10 based on previous regional data), and d denotes the margin of error (5% or 0.05).

Substituting these values into the formula yields $n = (1.96&sup2; &times; 0.10 &times; 0.90) / 0.0025 = 138.3$. Accordingly, the minimum required sample size was 139. However, 433 clinically diagnosed PTB cases were included to enhance statistical power, of which 40 cases were identified as LLTB and analyzed in detail for radiological-microbiological correlation.

Lower lobe classification and inclusion criteria

From the total PTB cohort, patients showing predominant lower lobe involvement formed the subset defined as LLTB. These cases were identified based on radiological findings (chest radiograph and HRCT), which showed dominant disease in the lower lung zones, confirmed by microbiological tests. An arbitrary horizontal line was drawn across the hila on posteroanterior (PA) chest radiographs to delineate upper and lower lung fields. The lower lung zones included the lingula (left lung), the middle lobe (right lung), and the lower lobes.

Cases with isolated pleural effusion, pleural thickening, or combined upper and lower lobe involvement without predominant lower lobe changes were excluded. Patients with known malignancies or other non-tubercular lung diseases were also excluded. For comparative analysis, the remaining patients without predominant lower lobe involvement were considered the non-lower lobe TB (non-LLTB) group. The clinical, demographic, and radiological characteristics of both groups were compared using appropriate statistical tests.

Data collection and imaging techniques

A computer-assisted search of the institutional radiology database was performed to locate reports of computed tomography (CT) thorax and PA chest radiographs that confirmed diagnoses of PTB. The study encompassed all PTB cases characterized by substantial lower lobe involvement, as established through radiological imaging and microbiological analysis. All PTB cases were analyzed descriptively, while LLTB cases underwent detailed correlations based on radiological and microbiological findings. CT scans were conducted with a 16-slice multidetector computed tomography (MDCT) scanner (GE Brivo 385, GE Healthcare, Milwaukee, USA) [10]. The imaging parameters for the scout film were established at 120 kV, 10 mA, and a window width/window level (WW/WL) of 500/50. The diagnostic scan parameters were 120 kV, 120 mA, a slice thickness of 1.25 mm, a reconstruction interval of 20 mm, and a rotation duration of one second. Two radiologists, each possessing over five years of proficiency in thoracic imaging, independently assessed the chest radiographs and HRCT images. LLTB was detected in the imaging patterns, and interpretative disagreements were reconciled through consensus. The assessed radiographic parameters included the extent and distribution of pulmonary involvement, types of opacities, presence of nodules, calcifications, lymphadenopathy, patterns of contrast enhancement, and other abnormalities such as pleural effusion, bronchiectasis, and fibrosis. All images were assessed utilizing lung window settings to enhance parenchymal visibility.

Microbiological analysis

The diagnosis of PTB was confirmed using a combination of clinical evaluation, radiographic evidence, and microbiological analysis. The microbiological techniques employed included Ziehl-Neelsen (ZN) staining for AFB on direct sputum smears, culture on Löwenstein-Jensen (LJ) medium (HiMedia Laboratories Pvt. Ltd., Mumbai, India) for *Mycobacterium tuberculosis* proliferation, and the GeneXpert MTB/RIF assay (Cepheid, Sunnyvale, CA, USA) for the detection of *Mycobacterium tuberculosis* DNA and rifampicin resistance in specific instances. Sputum-negative PTB was characterized by three consecutive negative AFB smears and cultures, confirmed by radiological evidence and an absence of clinical improvement following broad-spectrum antibiotic treatment.

HIV testing and treatment

After obtaining separate informed consent, all PTB patients were offered HIV testing by the Centers for Disease Control and Prevention (CDC) guidelines [11]. Upon confirmation of PTB, patients were detected on a standard short-course anti-tubercular therapy (ATT) regimen following the World Health Organization's (WHO) recommendation [12], thereby ensuring adherence to established treatment protocols.

Statistical analysis

Statistical analysis was performed using IBM SPSS Statistics for Windows, Version 29 (Released 2023; IBM Corp., Armonk, New York). Descriptive data were analyzed using means and standard deviations. Categorical variables were compared using chi-square tests, and continuous variables using independent t-tests. Sensitivity and specificity analyses were performed to evaluate the diagnostic accuracy of radiographic and HRCT findings in detecting LLTB. Meanwhile, correlation analysis was used to assess the relationship between microbiological findings, radiographic patterns, and HRCT features.

## Results

Table 1 presents the gender distribution of diagnosed, radiologically positive, and LLTB cases. Among the 433 clinically diagnosed cases, 238 (55%) were male and 195 (45%) were female. Of these, 181 cases were radiologically confirmed as TB, with 100 (55.2%) males and 81 (44.8%) females. Among the 40 cases identified with LLTB, 24 (60.0%) were male and 16 (40.0%) were female.

**Table 1:** Gender distribution of clinically diagnosed, radiologically positive, and lower lobe tuberculosis cases

Parameters	Male N (%)	Female N (%)	Total N (%)
Clinically diagnosed cases	238 (55.0%)	195 (45.0%)	433 (100.0%)
Radiologically positive tuberculosis cases	100 (55.2%)	81 (44.8%)	181 (100.0%)
Lower lobe tuberculosis cases	24 (60.0%)	16 (40.0%)	40 (100.0%)

Among the 74 patients with radiological abnormalities, the most common feature was patchy opacities seen in 37 (50.0%) cases, followed by homogeneous consolidation in 31 (41.9%), cavitation in 14 (18.9%), nodular opacities in nine (12.2%), and para-pneumonic effusion in eight (10.8%) cases. Regarding diagnostic methods, the sputum smear test for AFB was positive in 33 (18.2%) cases, bronchoalveolar lavage (BAL) was AFB-positive in 12 (6.6%) cases, CT findings were suggestive of TB in 16 (8.8%) cases, and nine (5.0%) patients had sputum-negative but unresolving pneumonia that responded to ATT. In terms of clinical presentation, cough with scanty expectoration was reported in 77 (42.5%) patients, mild to moderate fever in 55 (30.4%), weight loss in 42 (23.2%), hemoptysis in 11 (6.1%), and pleuritic chest pain in eight (4.4%) patients (Table 2).

**Table 2:** Radiological features, diagnostic methods, and clinical symptoms of radiologically positive tuberculosis (N=181) AFB: acid-fast bacilli; TB: tuberculosis; CT: computed tomography

Variables	Number of Cases N (%)
Radiological features (N=74)	
Patchy opacities	37 (50.0%)
Homogeneous consolidation	31 (41.9%)
Cavitation	14 (18.9%)
Nodular opacities	9 (12.2%)
Para-pneumonic effusion	8 (10.8%)
Diagnostic methods	
Sputum smear for AFB positive	33 (18.2%)
Bronchoalveolar lavage positive for AFB	12 (6.6%)
CT findings suggestive of TB	16 (8.8%)
Sputum negative but unresolving pneumonia (responded to treatment)	9 (5.0%)
Clinical symptoms	
Cough with scanty expectoration	77 (42.5%)
Mild to moderate fever	55 (30.4%)
Weight loss	42 (23.2%)
Pleuritic pain	8 (4.4%)
Hemoptysis	11 (6.1%)

Table 3 shows the gender-wise distribution of radiological findings among 40 LLTB cases, including 24 males and 16 females. The most common radiological feature was patchy opacities, seen in 20 (50%) patients, comprising 13 (54.1%) males and seven (43.7%) females. Homogeneous consolidation was observed in 17 (42.5%) cases, with 11 (45.8%) males and six (37.5%) females. Cavitation was present in eight (20%) patients, including five (20.8%) males and three (18.75%) females. Nodular opacities were noted in five (12.5%) cases, equally distributed among males (three cases, 12.5%) and females (two cases, 12.5%). Para-pneumonic effusion was identified in four patients (10%), with three (12.5%) males and one (6.25%) female.

**Table 3:** Gender-wise distribution of radiological findings in lower lobe tuberculosis (LLTB) cases (n=40) Data are presented in the N (%) format.

Radiological Features	Male (N=24)	Female (N=16)	Total (N=40)
Patchy opacities	13 (54.1%)	7 (43.7%)	20 (50%)
Homogeneous consolidation	11 (45.8%)	6 (37.5%)	17 (42.5%)
Cavitation	5 (20.8%)	3 (18.75%)	8 (20%)
Nodular opacities	3 (12.5%)	2 (12.5%)	5 (12.5%)
Para-pneumonic effusion	3 (12.5%)	1 (6.25%)	4 (10%)

Table 4 compares LLTB cases (n = 40) with non-LLTB cases (n = 393) across demographic, clinical, and radiological variables. The mean age was higher in the LLTB group (48 ± 12 years) than in the non-LLTB group (42 ± 14 years), showing a statistically significant difference (t = 2.01, p = 0.045). Males accounted for 24 (60%) in the LLTB and 214 (54.6%) in the non-LLTB group, with no significant difference. Clinical features such as cough with expectoration (19 (47.5%) vs. 58 (41.3%)), fever (14 (35%) vs. 41 (29.1%)), weight loss (10 (25%) vs. 32 (22.7%)), hemoptysis (three (7.5%) vs. eight (5%)), and pleuritic chest pain (two (5%) vs. six (4.3%)) were slightly more frequent in the LLTB group but without statistical significance (p > 0.05). Radiological findings, including patchy opacities (20 (50%) vs. 57 (43.5%)), homogeneous consolidation (17 (42.5%) vs. 50 (38.2%)), cavitation (eight (20%) vs. 23 (17.6%)), nodular opacities (five (12.5%) vs. 14 (10.7%)), and para-pneumonic effusion (four (10%) vs. 12 (9.2%)), were similarly distributed between groups. However, HRCT suggestive of TB was significantly higher in LLTB cases (eight (20%) vs. 32 (8.2%)) (χ² = 5.64, p = 0.018), indicating its diagnostic relevance in atypical presentations.

**Table 4:** Comparative clinical and radiological characteristics of LLTB and non-LLTB t: t-test c: chi-square *: significant p-value P-values less than 0.05 were considered significant LLTB: lower lobe tuberculosis; AFB: acid-fast bacilli; HRCT: high-resolution computed tomography; TB: tuberculosis

Variables	LLTB (N=40)	Non-LLTB (N=393)	Statistical Test	p-value
Demographics				
Mean age (years)	48 ± 12	42 ± 14	2.01^t^	0.045*
Sex/male	24 (60%)	214 (54.6%)	0.49^c^	0.49
Clinical features				
Cough with expectoration	19 (47.5%)	58 (41.3%)	0.47^c^	0.49
Mild to moderate fever	14 (35%)	41 (29.1%)	0.51^c^	0.47
Weight loss	10 (25%)	32 (22.7%)	0.11^c^	0.74
Pleuritic chest pain	2 (5%)	6 (4.3%)	0.04^c^	0.85
Hemoptysis	3 (7.5%)	8 (5%)	0.41^c^	0.52
Sputum AFB positive	9 (22.5%)	67 (17%)	0.77^c^	0.38
Radiological features				
Patchy opacities	20 (50%)	57 (43.5%)	0.61^c^	0.43
Homogeneous consolidation	17 (42.5%)	50 (38.2%)	0.24^c^	0.62
Cavitation	8 (20%)	23 (17.6%)	0.13^c^	0.72
Nodular opacities	5 (12.5%)	14 (10.7%)	0.09^c^	0.76
Para-pneumonic effusion	4 (10%)	12 (9.2%)	0.03^c^	0.87
HRCT suggestive of TB	8 (20%)	32 (8.2%)	5.64^c^	0.018*

Table 5 summarizes the correlation of radiological features with microbiological and CT findings in LLTB. Cavitation showed the strongest correlation with sputum smears (r = 0.80) and 90% specificity among the radiological features. Patchy opacities showed strong correlations across all diagnostic methods, with a sputum smear correlation of 0.75, a BAL correlation of 0.65, and a CT correlation of 0.70. Homogeneous consolidation and nodular opacities also demonstrated moderate correlations, with specificity values ranging from 82% to 88%. Para-pneumonic effusion had the lowest correlation values but the highest specificity in sputum smear (95%) and BAL (93%). Cavitation and patchy opacities were the most reliable radiological indicators, showing strong associations with microbiological and CT findings.

**Table 5:** Correlation of radiological features with microbiological and computed tomography findings in lower lobe tuberculosis

Radiological Features	Sputum Smear			Bronchoalveolar Lavage			Computed Tomography		
	Spearman's correlation r	Sensitivity %	Specificity	Spearman's correlation r	Sensitivity %	Specificity	Spearman's correlation r	Sensitivity %	Specificity
Patchy opacities	0.75	72.0%	85.0%	0.65	62.5%	80.0%	0.7	66.7%	83.3%
Homogeneous consolidation	0.72	71.4%	88.0%	0.62	62.5%	82.0	0.68	58.8%	84.0%
Cavitation	0.80	70.0%	90.0%	0.70	66.7%	85.0%	0.75	66.7%	88.0%
Nodular opacities	0.68	71.4%	92.0%	0.60	60.0%	90.0%	0.65	66.7%	85.0%
Para-pneumonic effusion	0.65	60.0%	95.0%	0.58	66.7%	93.0%	0.72	75.0%	91.0%

## Discussion

TB continues to pose a significant public health challenge worldwide. While PTB predominantly affects the upper lobes, involvement of the lower lobes presents diagnostic difficulties due to atypical clinical and radiological features. Comprehensive characterization of LLTB is essential for improving early detection and management.

The present study demonstrated a consistent male predominance across all TB categories examined: clinically diagnosed, radiologically confirmed, and LLTB cases. Among the 433 clinically diagnosed patients, 55% were male. This pattern persisted in the radiologically confirmed group (55.2%) and the LLTB subset (60%). These findings corroborate global epidemiological data reported by Horton et al. [13] and Akhtar et al. [14], who attributed higher TB prevalence in males to increased occupational exposure, tobacco use, alcohol consumption, and possible biological differences in immune response.

Clinically, cough with minimal expectoration was the most frequently reported symptom, observed in 42.5% of radiologically confirmed cases. This aligns with previous studies by Alaguraja et al. [15] and Yadav et al. [16], which identified chronic cough as a hallmark symptom of pulmonary TB. Fever was present in 30.4% of patients, reflecting a systemic inflammatory response consistent with findings by Dakappa et al. [17]. Weight loss, noted in 23.2% of cases, underscores the metabolic and cachectic effects of chronic infection, as described by Kalva et al. [10]. Hemoptysis occurred in 6.1%, likely secondary to cavitary lesions, concordant with observations by Kaur et al. [18]. Pleuritic chest pain was the least common symptom, indicating limited pleural involvement, as also reported by Vorster et al. [19].

Microbiologically, sputum smear positivity for AFB was documented in only 18.2% of cases, highlighting the substantial burden of smear-negative TB, consistent with findings by Seni et al. [20]. BAL contributed to diagnosis in 6.6% of patients, demonstrating its utility in sputum-scarce or smear-negative cases, in agreement with Ahmad et al. [21]. CT imaging aided diagnosis in 8.8% of cases, particularly in the absence of microbiological confirmation, reinforcing the role of imaging in atypical presentations such as LLTB, as emphasized by Caliskan et al. [22].

Radiologically, patchy opacities were the predominant finding (50.0%), frequently resembling bacterial pneumonia. This underscores the importance of clinicopathological correlation, as highlighted by Veske et al. [23]. Homogeneous consolidation (41.9%) overlapped similarly with the radiographic features of community-acquired pneumonia, complicating the differential diagnosis [22]. Cavitation was observed in 18.9% of cases, indicative of advanced or chronic disease stages and an increased transmission risk, consistent with prior reports [21-23]. Nodular opacities (12.2%) likely represent endobronchial spread, as described by Bhalla et al. [24]. Para-pneumonic effusion was identified in 10.8%, suggesting pleural involvement, aligning with findings by Radhakrishnan et al. [25].

The prevalence of LLTB in this cohort was 9.2% (40 out of 433 cases), comparable to earlier studies from India. For instance, research from Madhya Pradesh reported a 10% prevalence of LLTB, with consolidation as the most common radiographic pattern (56.7%), followed by cavitation (24.7%) and right-sided predominance [26]. Another study from Bangalore, involving 20 LLTB cases, described patchy opacities (45%), homogeneous consolidation (35%), and cavitation (15%) as principal imaging features [27]. Our findings of patchy opacities (50%), consolidation (41.9%), and cavitation (18.9%) are largely concordant, although a higher male predominance (60%) was noted, compared to some regional reports that indicate a female predominance [26,27]. These similarities support the external validity of our results, while variations may reflect demographic or geographic differences.

Further subgroup analysis demonstrated that cavitation and patchy opacities exhibited the strongest correlations with microbiological and HRCT findings, particularly sputum smear positivity (correlation coefficients r = 0.80 and r = 0.75, respectively). These radiological features emerged as the most reliable indicators for diagnosing LLTB. Although para-pneumonic effusion was less frequent, it showed high specificity but weaker correlation, suggesting its diagnostic value when present but limited consistency. These results suggest that LLTB frequently presents with non-specific radiological findings and lower rates of sputum positivity, which may delay diagnosis. An integrated approach combining clinical evaluation, microbiological testing, and advanced imaging modalities such as HRCT is crucial for prompt and accurate diagnosis, especially in smear-negative or atypical cases. Given the diagnostic challenges and clinical significance of LLTB, increased awareness and utilization of imaging support are essential for early detection and effective management.

Limitations

This study has several limitations that should be considered while interpreting the findings. The relatively small sample size of 40 microbiologically and radiologically confirmed LLTB cases limits the statistical power and generalizability of the results. As the study was conducted at a single tertiary care center, the diagnostic and treatment approaches may have been influenced by local institutional protocols, which could limit the applicability of the findings to other clinical settings. Although experienced radiologists independently reviewed the imaging, the possibility of interobserver variation in interpretation cannot be entirely excluded. Furthermore, microbiological confirmation could not be established in all cases, and diagnosis in a subset of patients relied on clinicopathological correlation, which may have introduced diagnostic uncertainty. The lack of longitudinal follow-up data precluded the assessment of treatment response and long-term outcomes, which are essential for evaluating the prognostic significance of specific radiological patterns.

Clinical and diagnostic significance

The significant prevalence of patchy opacities and homogenous consolidation suggests a frequent misidentification of LLTB as bacterial pneumonia. This misunderstanding may delay diagnosis and lead to the improper use of antibiotics. Consequently, integrating microbiological, radiological, and clinical evaluations is crucial for early and precise diagnosis. Cavitation in approximately 19% of instances highlights the importance of early identification in managing disease prognosis. Furthermore, the significance of BAL and HRCT in detecting smear-negative TB cases underscores the necessity for multimodal diagnostic approaches.

## Conclusions

This study highlights that LLTB presents distinct clinical and radiological features that differ from typical PTB. It is more commonly seen in male patients and often mimics bacterial pneumonia due to its imaging patterns, which include patchy opacities and homogeneous consolidation. Although less frequently observed, cavitation exhibits a strong association with microbiological confirmation and serves as a reliable diagnostic indicator. The tendency of LLTB to yield negative sputum results makes diagnosis more challenging, emphasizing the value of HRCT and BAL. A combined approach using clinical, radiological, and microbiological assessments is essential for accurate and timely diagnosis of this atypical form of TB.

## References

[1] (2025). Global tuberculosis control: surveillance, planning, financing. https://www.who.int/publications/i/item/9241563141.

[2] Uys P, Marais BJ, Johnstone-Robertson S, Hargrove J, Wood R (2011). Transmission elasticity in communities hyperendemic for tuberculosis. Clin Infect Dis.

[3] Raniga S, Parikh N, Arora A, Vaghani M, Vora PA, Vaidya V (2006). Is HRCT reliable in determining disease activity in pulmonary tuberculosis?. Indian J Radiol Imaging.

[4] Khan ZU, Muhammad A, Ullah I, Mir A (2017). Yield of direct versus concentrated sputum microscopy for diagnosis of pulmonary tuberculosis. Khyber J Med Sci.

[5] Woodring JH, Vandiviere HM, Fried AM, Dillon ML, Williams TD, Melvin IG (1986). Update: the radiographic features of pulmonary tuberculosis. AJR Am J Roentgenol.

[6] Hatipoğlu ON, Osma E, Manisali M (1996). High resolution computed tomographic findings in pulmonary tuberculosis. Thorax.

[7] Tozkoparan E, Deniz O, Ciftci F (2005). The roles of HRCT and clinical parameters in assessing activity of suspected smear negative pulmonary tuberculosis. Arch Med Res.

[8] Ors F, Deniz O, Bozlar U (2007). High-resolution CT findings in patients with pulmonary tuberculosis: correlation with the degree of smear positivity. J Thorac Imaging.

[9] Naseem A, Saeed W, Khan S (2008). High resolution computed tomographic patterns in adults with pulmonary tuberculosis. J Coll Physicians Surg Pak.

[10] Kalva J, Babu SP, Narasimhan PB (2023). Predictors of weight loss during the intensive phase of tuberculosis treatment in patients with drug-susceptible pulmonary tuberculosis in South India. J Public Health (Oxf).

[11] Branson BM, Handsfield HH, Lampe MA, Janssen RS, Taylor AW, Lyss SB, Clark JE (2006). Revised recommendations for HIV testing of adults, adolescents, and pregnant women in health-care settings. MMWR Recomm Rep.

[12] World Health Organization (2024). Treatment of Tuberculosis: Guidelines, 4th Edition. https://www.ncbi.nlm.nih.gov/books/NBK138748/.

[13] Horton KC, MacPherson P, Houben RM, White RG, Corbett EL (2016). Sex differences in tuberculosis burden and notifications in low- and middle-income countries: a systematic review and meta-analysis. PLoS Med.

[14] Akhtar S, White F, Hasan R (2007). Hyperendemic pulmonary tuberculosis in peri-urban areas of Karachi, Pakistan. BMC Public Health.

[15] Alaguraja S, Aruna G, Subbarao S, Bharat K (2022). TB and other chest infections. Lung India.

[16] Yadav P, Gupta AK, Gautam AK, Kumar A, Priyadarshi S, Srivastav DK (2022). Clinico-bacteriological profile of community-acquired pneumonia patients at tertiary care center of North India. Indian J Respir Care.

[17] Dakappa PH, Rao SB, B G, Bhat GK, Mahabala C (2019). Unique temperature patterns in 24-h continuous tympanic temperature in tuberculosis. Trop Doct.

[18] Kaur H, Pandhi N, Kajal NC (2022). A prospective study of the clinical profile of hemoptysis and its correlation with radiological and microbiological findings. Int J Mycobacteriol.

[19] Vorster MJ, Allwood BW, Diacon AH, Koegelenberg CF (2015). Tuberculous pleural effusions: advances and controversies. J Thorac Dis.

[20] Seni J, Kidenya BR, Obassy E (2012). Low sputum smear positive tuberculosis among pulmonary tuberculosis suspects in a tertiary hospital in Mwanza, Tanzania. Tanzan J Health Res.

[21] Ahmad M, Ibrahim WH, Sarafandi SA (2019). Diagnostic value of bronchoalveolar lavage in the subset of patients with negative sputum/smear and mycobacterial culture and a suspicion of pulmonary tuberculosis. Int J Infect Dis.

[22] Caliskan T, Ozkisa T, Aribal S, Kaya H, Incedayi M, Ulcay A, Ciftci F (2014). High resolution computed tomography findings in smear-negative pulmonary tuberculosis patients according to their culture status. J Thorac Dis.

[23] Veske NŞ, Onur ST, Abalı H (2023). Differentiating pulmonary tuberculosis from bacterial pneumonia: the role of inflammatory and other biomarkers. Istanbul Med J.

[24] Bhalla AS, Goyal A, Guleria R, Gupta AK (2015). Chest tuberculosis: radiological review and imaging recommendations. Indian J Radiol Imaging.

[25] Radhakrishnan P, Mathanraj S (2020). Role of pleural fluid C-reactive protein in the aetiological diagnosis of exudative pleural effusion. J Clin of Diagn Res.

[26] Singh SK, Tiwari KK (2015). Clinicoradiological profile of lower lung field tuberculosis cases among young adult and elderly people in a teaching hospital of Madhya Pradesh, India. J Trop Med.

[27] Kavitha K, Geetha SH, Dayananda Kumar R, Shirbur P (2024). Microbiological, radiographic and HRCT correlation in lower lobe tuberculosis. Int J Pharm Clin Res.

